# A new simple morphology‐based risk score is prognostic in stage I/II colon cancers

**DOI:** 10.1002/cam4.737

**Published:** 2016-05-11

**Authors:** Bruno Märkl, Maximilian Märkl, Tina Schaller, Patrick Mayr, Gerhard Schenkirsch, Bernadette Kriening, Matthias Anthuber

**Affiliations:** ^1^ Institute of PathologyKlinikum AugsburgAugsburgGermany; ^2^ Clinical and Population‐Based Cancer Registry AugsburgKlinikum AugsburgAugsburgGermany; ^3^ Department of Visceral‐ and Transplantation SurgeryKlinikum AugsburgAugsburgGermany

**Keywords:** Colon Cancer, Histomorphology, Prognosis, Risk Stratification, Stage I/II

## Abstract

A portion of stage I/II colon cancers (10–20%) exhibit an adverse clinical course. The administration of adjuvant chemotherapy is recommended only in certain high‐risk situations. However, these risk factors recently failed to predict benefit from adjuvant therapy. We composed a new morphology‐based risk score that includes pT1/2 versus 3/4 stage, vascular or lymphovascular invasion, invasion type according to Jass, tumor budding and paucity (less than two) of lymph nodes larger than 5 mm. The occurrence of each of these factors accounts for one point in the score (Range 0–5). This score was evaluated in a retrospective study that included 301 cases. The overall survival differed significantly between the three groups with median survival times of 103, 90, and 48 months, respectively. Multivariable analysis revealed morphology‐based risk—high risk and low risk—as the sole independent factors for the prediction of death. Morphology‐based risk scoring was superior to microsatellite status and NCCN risk stratification. This method identifies a group of patients that comprises 18% of the stage II cases with an adverse clinical course. Further studies are necessary to confirm its prognostic value and the possible therapeutic consequences.

## Introduction

Colon cancer is one of the leading malignant diseases worldwide. In 2014, there were approximately 100,000 new colon cancer cases expected in the US. As in other types of cancer, the prognosis strongly depends on the stage of the disease. In addition to the Dukes classification, the UICC scheme gained general acceptance and is widely used for prognosis estimation and therapy stratification 1. Healing from cancer is attempted in all cases without distant metastases, which belong to UICC stages I–III. However, a curative therapeutic approach in metastatic disease is possible only in a certain number of cases. Among those cases, the node negative cases are classified as stage I or II, depending on the infiltration depth. These patients are known to have an excellent prognosis 2. Adjuvant chemotherapy promises only a small benefit in these cases 3 and is therefore restricted to particular risk situations 2. Nevertheless, approximately 10% to 20% of colon cancer cases show an adverse clinical course. To date, there is no generally accepted diagnostic tool available that could predict which of those cases are prone to developing progressive disease. Many authors suggested false node negativity and stage migration to explain the aggressive behavior of certain stage II cancers 4, 5. However, we and others recently could show that nodal understaging is most likely an overestimated problem and occurs only in a small portion of cases. These reports could disprove that the prognostic strength of the UICC staging could be heightened by improving lymph node retrieval and detection of lymph node metastases. The NCCN defined several additional factors to identify patients at an increased risk for progressive disease in stage II colorectal cancers 2. These factors include T4 stage, inadequate lymph node harvest, emergency situation, and obstruction. Several attempts have been made to establish risk stratification systems based on molecular testing. To date, only testing for microsatellite instability received a recommendation by the NCCN. Gene microarrays were developed in analogy to their use in breast cancer. These tests could be shown to be prognostically independent of the established factors of the TNM system 2, 6, 7. Nevertheless, the evidence for these tests seems insufficient for a general recommendation. The drawbacks of molecular testing are the high costs, and the need for fresh tumor tissue for the ColoPrint^®^ test. A promising concept to distinguish prognostically different subgroups of colon cancer has been introduced by Galon et al. that is based on the evaluation of the immune response against the tumor. An immune score is calculated by counting the number of CD3‐ and CD8‐positive T‐lymphocytes in the tumor center and at the invasion front. A large international prospective multicenter trial is currently underway to evaluate this concept 8, 9.

As an alternative approach to sophisticated molecular tests, we hypothesized that a risk score based on a panel of histological features could provide additional information to predict the clinical course of stage I/II colorectal cancer more precisely than UICC staging alone. It was our goal to establish a score that it is easy to evaluate without the need for extra time and costs. The selection of the factors was based on the experiences of different previously performed studies 10, 11, 12 and on the idea of including factors that represent various aspects of tumor progression. For this morphology‐based risk (MBR) score, we included T‐stage and vascular and lymphovascular invasion as established factors of the TNM classification system. Additionally, tumor budding and invasion type according to Jass 13 were included. The fifth factor is the paucity of intermediate to large sized lymph nodes, which recently has been proposed as a new prognostic factor in colon cancer. Paucity in this context is defined as less than two lymph nodes with a diameter larger than 5 mm. We could demonstrate that the number of lymph nodes larger 5 mm (LN5) is associated with lymph node count and outcome 10. Moreover, we reported an association between this factor and lymphocytic tumor infiltration, which indicate that LN5 could serve as a surrogate marker for immune response.

## Materials and Methods

### Patients

Cases from 2002 to 2005 and from 2007 to 2010 were retrieved from our files. The cases from the first period belong to an era where conventional lymph node dissection was performed. Since 2007, the methylene blue‐assisted lymph node dissection technique was routinely used. The cases from 2006 were excluded because the specimens were dissected in very different ways and by a high number of differently qualified staff (pathologists, residents, technicians). The study cases were retrieved by a search within the administrative database (Nexus AG, Frankfurt am Main, Germany) of the Institute of Pathology.

**Figure 1:**
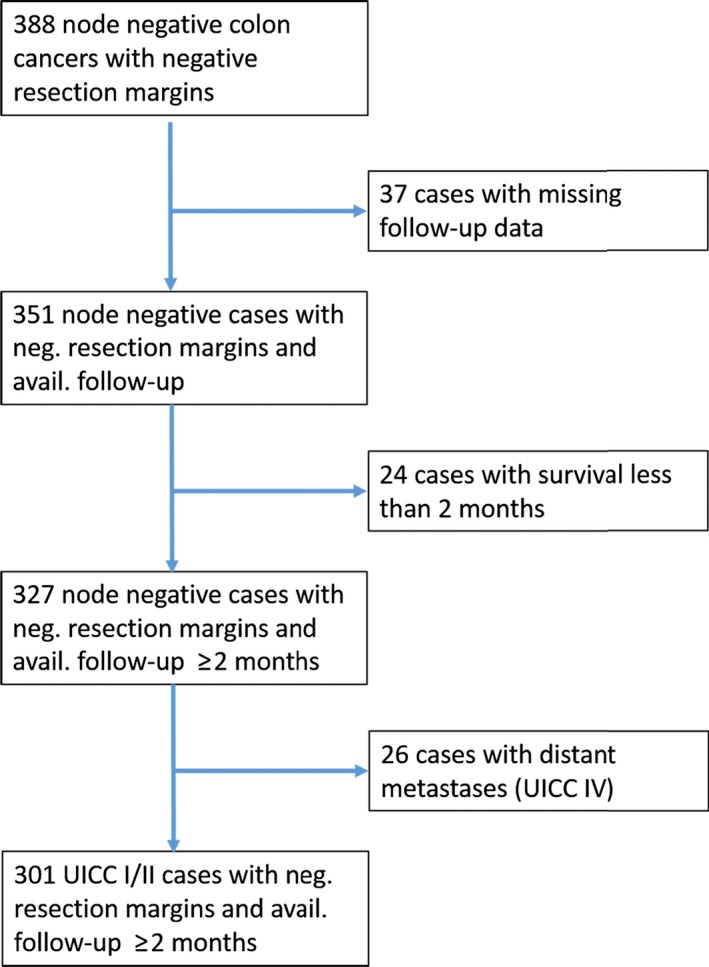
Selection of cases after research for node negative colon cancers within the files of the Institute of Pathology of the Klinikum Augsburg.

Inclusion criteria were stage I/II colon cancers, curative intention, and a minimal follow‐up period of two months. Criteria of exclusion were positive resection margins and clinical or histologic evidence of metastatic disease. Follow‐up data were provided by the Clinical and Population based Cancer Registry Augsburg. This register is responsible for the region of Swabia with 1.8 million inhabitants. It receives cancer‐ and patient‐related information from all hospitals and from practicing oncologists. Importantly, it can match their data with the deaths in the region. Additionally, the clinical information system and the files of the pathology department were screened for more case‐related information. A flowchart that outlines the patient selection is given in Figure 1. The study was performed according to the national rules and approved by the internal review board of the Klinikum Augsburg.

### Histopathological evaluation, morphometry, and immunohistochemistry

**Figure 2:**
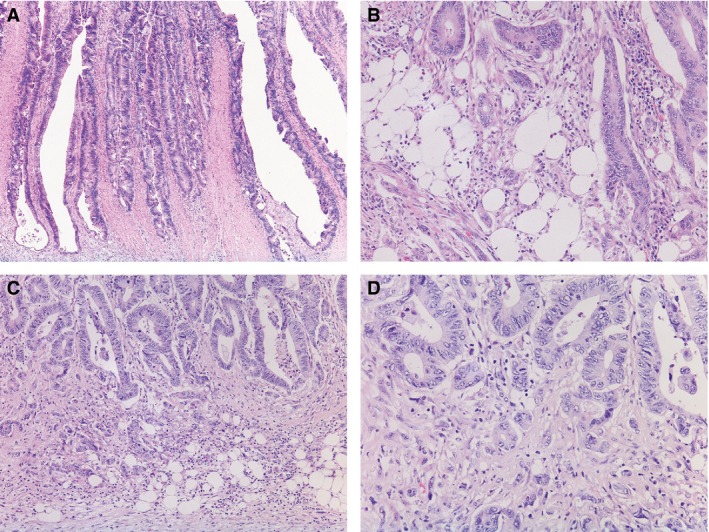
(A) HE, 16x; Colon carcinoma with infiltrative invasion pattern. The muscularis propria is dissected by streaming atypical tumor glands. (B) HE, 50x; Same case with infiltration of the mesenteric fat with relatively little stromal response. (C) HE, 16x; Invasion front of a colon cancer with extensive tumor budding. (D) HE, 100x; Same case with higher magnification.

Factors and scoring of the MBR‐score are shown in Table 1. All histopathological factors of the MBR‐score were evaluated on hematoxylin‐ and eosin‐stained slides. No immunohistochemistry was performed, except for the evaluation of the mismatch repair genes. Tumor budding was defined as positive in cases where more than 30 tumor buds of ≤ 5 cells were identified at the invasion front in a field of 1.22 mm² (Fig. 2A). The invasion type (Fig. 2B) was evaluated according to the definition by Jass. All cases were re‐evaluated by two independent pathologists (BM and TS) who read one tumor slide blinded to the original reports and the case‐related information. In cases of discrepancy, a consensus diagnosis on a double‐headed microscope was made using additional slides.

**Table 1:** MBR‐Score

Worse criteria	Number of adverse points	Risk
pT3/4 ‐Stage	0	low
Infiltrative invasion type		
Tumor budding	1–2	intermediate
Vascular invasion (V1 and/or L1)		
<2 LN5	3–6	high

For the comparison of the significance of MBR‐score with the established criteria of the NCCN guideline, the occurrence of one or more of the following criteria was classified as high‐risk situation: lymphatic/vascular invasion, tumor perforation (= pT4), poor lymph node harvest (<12 LNs), poor differentiation, and perineural invasion. Cases with positive or indeterminate resection margins were not included in the study as mentioned above 2.

The morphometric analysis of lymph nodes to determine the number of LN5‐nodes was performed using either a digital camera with a calibrated software system (progress C3, Jenoptik, Wetzlar, Germany) (93 cases) or a simple caliber with a 5 mm punch (208 cases), as described previously 10, 11.

In all cases where at least one of the Bethesda criteria regarding Lynch syndrome was fulfilled, microsatellite diagnostics, including comparative PCR analysis of the loci of the consensus panel and immunohistochemistry for the mismatch repair genes MLH1, MSH2, MSH6 and PMS2, were performed. Additionally, all cases with medullary features or mucinous carcinomas were immunohistochemically stained for MLH1. In these cases, grading depended on the microsatellite status. Moreover, MLH1 expression was evaluated in 127 additional cases of the cohort that were not suspicious for a lynch syndrome and had no mucinous or medullary differentiation to enrich the number of cases with known mismatch‐repair status. Immunostaining was performed as described previously 10.

### Outcome measurements and subgroups

Overall survival was chosen as the endpoint for the outcome comparison. It was defined as the time between operation and death. Comparisons were performed between the three MBR‐score groups (Table 1) in UICC stage I/II cancers and separately for each of the two stages. Further analysis was performed in 151 microsatellite stable cases to exclude confounding by microsatellite instability. Additional comparisons were performed between cancers without and with at least one NCCN‐risk factor.

### Statistics

Depending on the results of normality testing, metric values were compared using either the student's t‐test or the Mann–Whitney rank sum test. Dichotomous data were analyzed with the *χ*² test or Fisher's exact test, depending on the sample size. For the evaluation of interobserver agreements, Cohen's kappa coefficient was calculated. For the determination of the median follow‐up time, the method of Schemper and Smith was used 14. Kaplan–Meier curves were created, and log‐rank regression analysis were performed to compare overall and tumor‐related survival of the different groups. Multivariable analysis was performed using the Cox regression proportional hazards model. *P* < 0.05 were considered to be significant. All calculations were performed using the Sigma‐Plot 13.0 software package (Systat, Erkrath, Germany).

## Results

### Patients, study groups and interobserver agreement

We included a total of 301 cases. The low, intermediate, and high groups comprised of 64, 208, and 29 cases, respectively. The clinicopathological data are summarized in Table 2. The two significantly different factors are pT‐stage and location. MBR high‐risk cancers are all locally advanced pT3/4 cases that occur primarily in the left colon. These patients tend to be older (72 vs. 70 and 68 years; *P* = 0.11). From 17 tumors with known MMR‐status, 16 were classified as MMR proficient (94%). Comparison between the evaluation results from the two pathologists showed moderate agreement for the factors *vascular invasion* (*κ *= 0.41) and *invasion type* (*κ *= 0.56). Substantial agreement was achieved for the factors *tumor budding* (*κ *= 0.61), *lymphovascular invasion* (*κ *= 0.66) and *T‐stage* (*κ *= 0.75).

**Table 2:** Clinicopathological data

	Low *n* = 64	Intermediate *n* = 208	High *n* = 29	P‐Value
Mean Age ± SD	68 ± 10	70 ± 10	72 ± 12	*P* = 0.11
Age <50	3 (5%)	9 (4%)	2 (7%)	*P* = 0.83
Gender m: f	1: 0.7	1: 0.7	01:01	*P* = 0.78
Mean LN count ± SD	22 ± 14	21 ± 15	20 ± 13	*P* = 0.51
LN count <12	8 (8%)	39 (19%)	6 (21%)	*P* = 0.47
Conventional Adenocarcinoma	57 (89%)	176 (85%)	27 (93%)	
Mucinous type	5 (8%)	22 (11%)	1 (3%)	
Medullary type	1 (2%)	8 (4%)	1 (3%)	
Other Types	1 (2%)	2 (1%)	0 (0%)	*P* = 0.36^b^
pT1	13 (20%)	15 (7%)	0 (0%)	
pT2	51 (80%)	55 (26%)	0 (0%)	
pT3	0	132 (63%)	26 (90%)	
pT4	0	6 (3%)	3 (10%)	*P* = 0.001^c^
Low grade	55 (86%)	160 (77%)	21 (72%)	
High Grade	9 (14%)	48 (23%)	8 (28%)	*P* = 0.220
Right sided	43 (67%)	102 (49%)	10 (34%)	
Left sided	21 (33%)	106 (51%)	6 (21%)	*P* = 0.006
MSS^a^	30 (75%)	105 (86%)	16 (94%)	
MSI^a^	10 (25%)	17 (14%)	1 (6%)	*P* = 0.125
Chemotherapy adj.	0	15 (7%)	3 (10%)	*P* = 0.06
Chemotherapy—adj. and pal.	1 (2%)	24 (12%)	7 (24%)	*P* < 0.001

SD = standard deviation, LN = lymph node, MSS = microsatellite stabile, MSI = microsatellite instable.

a microsatellite stability data were available only in 179 cases.

b analysis compared conventional versus the three other categories,

c only the pT stages of the intermediate and the high‐risk groups were compared, adj. = adjuvant, pal. = palliative.

### The distribution of worse factors in the high‐risk group

**Figure 3:**
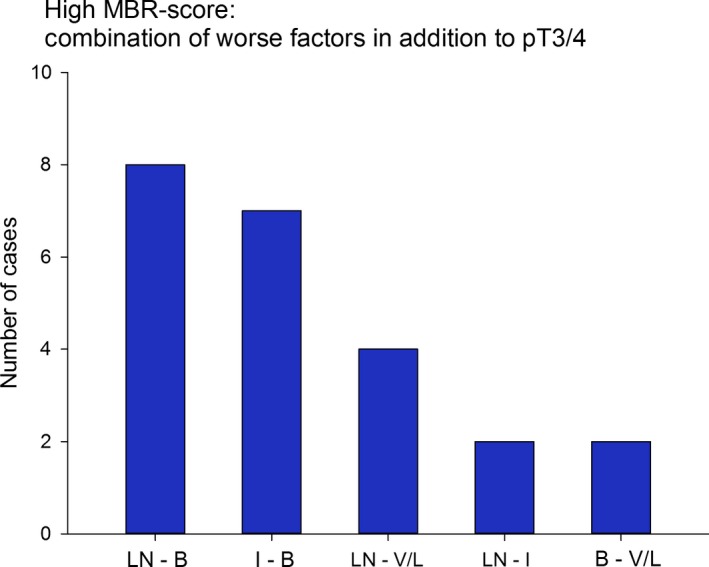
Distribution of worse factors in MBR score 3 cases. All cases showed an infiltration of the mesenteric fat or the serosal layer. LN = less than 2 lymph nodes were identified; B  =  Tumor budding; I = infiltrative invasion type; V/L vascular or lymphovascular invasion. The paucity of LN5 and tumor budding were the most frequent factors contributing to the high‐risk situation.

To identify the factors that contributed most frequently to a MBR high‐risk score, the different combinations of worse factors were counted. As mentioned above, all cases in the high‐risk group show an infiltration beyond the muscularis propria (pT3/4). The group of MBR high‐risk cases consists of 23 cases with a MBR score of 3, four cases with a MBR score of 4, and two cases with a MBR score of 5. The combination of the other worse risk factors in the MBR score 3 category is given in Table 1. Out of the 29 total cases classified as MBR high risk, tumor budding and LN5 classification were found in 23 (79%) and 18 (62%) cases, respectively. The most frequently found combination was pT3/4, tumor budding, and LN5vl (Fig. 3). This indicates that the latter two factors are of special importance in stage II cancers.

### MBR score‐related survival

**Figure 4:**
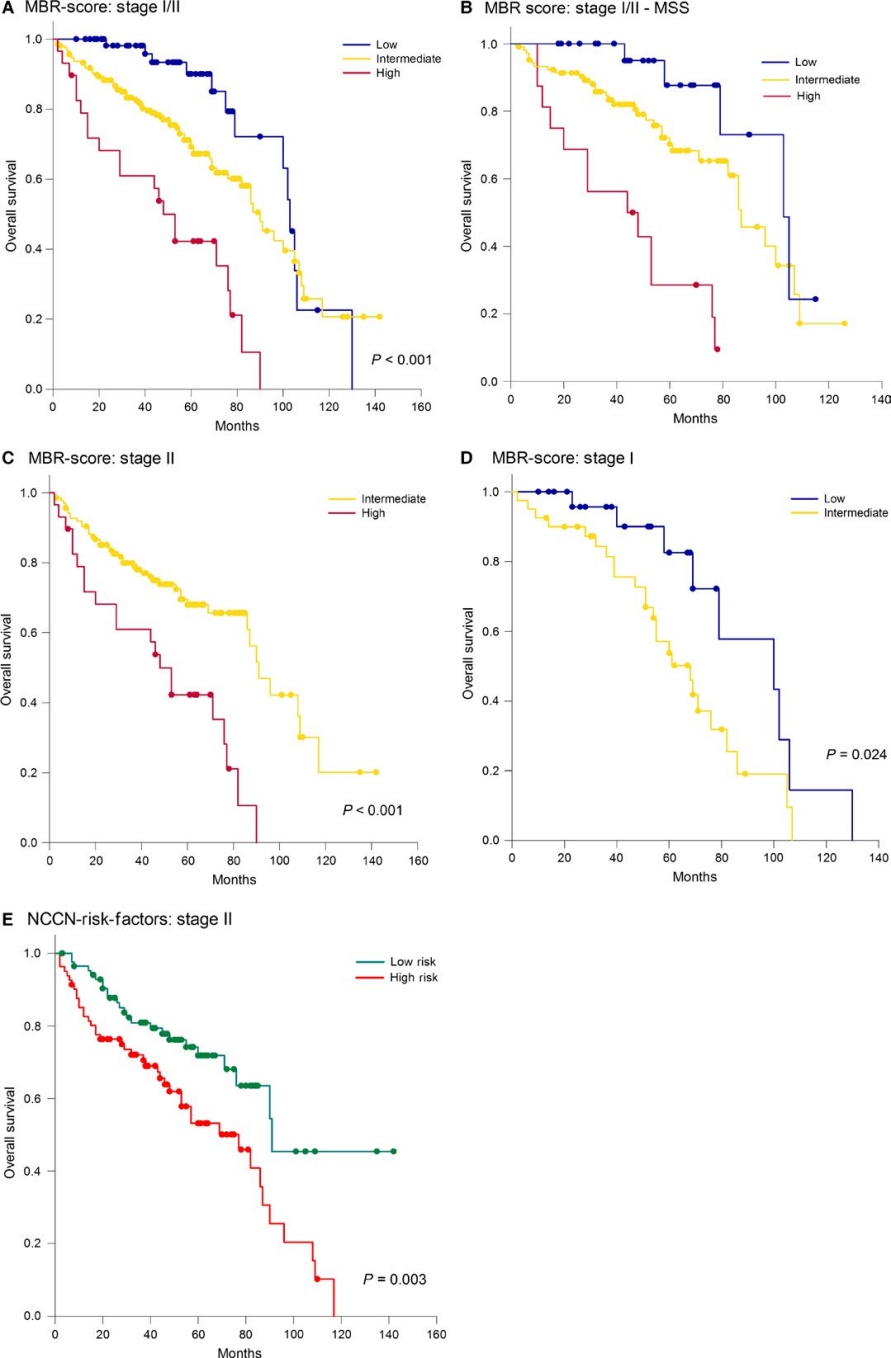
Overall survival analysis: (A) stratified according to MBR scoring including all cases (stage I and II), (B) stratified according to MBR scoring in the subgroup of microsatellite stable cases, (C) stratified according to MBR scoring restricted to stage II cases, (D) stratified according to MBR scoring restricted to stage II cases, (E) stratified according to occurrence of NCCN risk factors in stage II cases.

The median time for which the patients were followed was 60 months (range: 2–143 months). The classification of stage I/II colon cancers according to the MBR score reveals three highly significantly different prognostic groups (*P* < 0.001) (Fig. 4). The median overall survival times were 103 months (95% Confidence Interval (CI): 98–107 months), 90 months (CI: 79–101 months), and 48 months (CI: 37–59) for the low‐, intermediate‐, and high‐risk group, respectively (Fig. 4A). This finding held true when the analysis was restricted to stage II colon cancers with median survival times of 90 months (CI: 80–102) and 48 months (CI: 37–59 months), *P* < 0.001 (Fig. 4C) for the intermediate‐ and the high‐risk group, respectively. Note that the low‐risk group includes no stage II cancers as per definition. A subgroup analysis concerning stage I cancers showed that none of these cases belonged to the MBR high‐risk score. Nevertheless, the comparison of low‐ and intermediate‐risk cases revealed a significant worse clinical course of the intermediate‐risk group. The corresponding survival times were 100 months (CI: 50–150) and 68 months (CI: 52–84) for the low‐risk and the intermediate‐risk group, respectively (Fig. 4D).

### MSS stratified survival, comparison with NCCN risk stratification, and multivariable analysis

**Figure 5:**
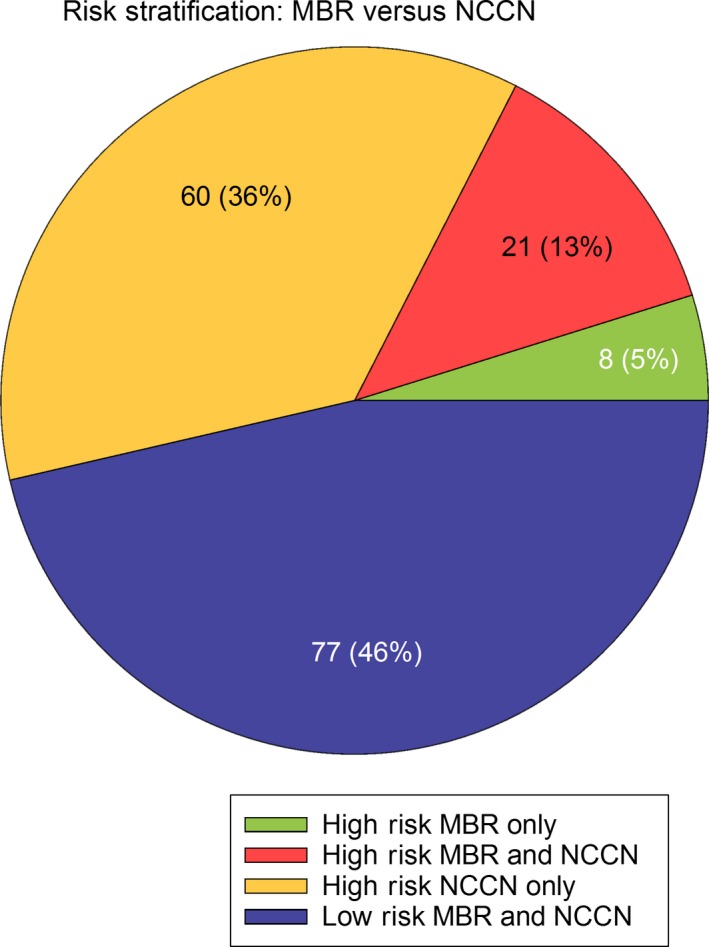
Distribution of risk groups in stage II colon cancers. Note. 18% of cases were graded high‐risk according to the MBR scoring while 49% of cases showed at least one NCCN risk factor.

MBR scoring was also prognostic when only cases with a known microsatellite stability status were analyzed. The corresponding median overall survival times were 103 months (CI: 80–126 months), 87 months (CI: 75–99 months), and 44 months (CI: 9–79 months), *P* < 0.001 for the low‐, intermediate‐, and high‐risk group, respectively. (Fig. 4B). The occurrence of at least one NCCN risk factor was associated with a highly significant worse outcome with median overall survival times of 91 months (CI: ± inf) versus 77 months (CI: 54–73 months), *P* = 0003 (Fig. 4E). However, the discrimination between the different prognostic groups is considerably weaker compared to MBR scoring. The distribution of risk stages based on NCCN and MBR score stratification is shown in Figure 5.

For a multivariable analysis of the whole study collective (stage I/II), the individual factors of the MBR‐score (pT stage, vascular invasion, lymphovascular invasion, invasion type, tumor budding, and LN5vl), the MBR stage, grading, and the microsatellite status were chosen because these factors are widely accepted as being prognostic. Cox regression proportional hazards model analysis revealed that MBR‐high risk and low risk were the sole independent factors for the prediction of death with hazard ratios of 4.12 (CI: 2.20–7.8) and 0.4 (CI: 0.18–0.9), respectively. Additional analysis performed in a subgroup of stage II cases with the additional inclusion of NCCN risk factor stratification also revealed that MBR high risk was the only predictive factor for overall survival (HR: 4.35; CI: 2.19–8.62).

## Discussion

Several attempts were made to improve the risk stratification in localized colon cancer. Under several developed multigene assays, Oncotype DX colon and ColoPrint gained the most recognition. Oncotype DX is a 12‐gene assay that proved to be independently prognostic (hazard rate 1.38) but not predictive when it was used on formalin‐fixed, paraffin‐embedded stage II cancers from the QUASAR trial 6. In contrast, fresh tissue is needed for the ColoPrint assay that includes 18 genes. Compared to other factors, this assay also independently discriminates between low‐ and high‐risk cases and was shown to be an independent predictor of distant metastases with a hazard ratio of 4.28 7. However, multigene assays are not recommended by the NCCN guideline 2015 because of lacking data that advocate adjuvant chemotherapy in high‐risk situations. 2. Based on an extensive hypoxia‐driven gene expression analysis, Dekervel et al. develop a six‐gene score that could be shown to be prognostic in stage II and III colon cancers 15. Molecular classifications have been introduced by Jass 16 and very recently with a very comprehensive approach by a large group of international experts 17. A different approach is the evaluation of the host's immune response to the tumor. Galon et al. developed an “Immuno” score that is based on the immunohistochemical evaluation of the densities of CD3‐ and CD8‐positive T‐lymphocytes in the tumor center and at the invasion front. The authors could show that its prognostic value is superior to conventional TNM staging 18.

With the exception of MSI testing, none of these innovative and sophisticated tests achieved broad clinical acceptance or even recommendation by relevant authorities yet 2. The benefit of adjuvant chemotherapy in localized colon cancer is notably small and is therefore restricted to high‐risk situations that have been defined by the NCCN 3, 19, 20, 21. However, a large study based on 24,847 patients from the Surveillance, Epidemiology, and End Results (SEER) database found no benefit for stage II cancers with or without these risk factors 22. Therefore, there was a demand to define more accurate risk factors to identify high‐risk patients who potentially could benefit more from adjuvant therapy 23, 24. Based on this situation, we had the idea to follow an apparently old‐fashioned approach to developing a test solely based on H&E morphology. In this retrospective study, we could show that the MBR score can discriminate among three prognostic groups of stage I/II colon cancers with a hazard ratio of 4.12 for the high‐risk group. This was independent of other risk factors and also held true in a subgroup of microsatellite cancers. The comparison of clincopathological factors showed a higher rate of left‐sided cancers, microsatellite stable and locally advanced cancers in the MBR high‐risk group (Table 2). These features could be an indication that the biology of these tumors differs fundamentally. Noteworthy, the mean age of the patients in the high‐risk group was higher than in the other groups (72 vs. 70 and 68 years) which is a potential bias concerning the overall survival analysis.

As mentioned before the basic selection criteria for the individual factors were easiness of its evaluation and their prognostic relevance. The score is composed of factors of the TNM system (pT stage and L/V classification), additional well‐investigated prognostic factors (invasion type and tumor budding), and a new prognostic factor (LN5 classification). The prognostic relevance of the infiltration depth and lymphovascular and vascular invasion has been confirmed in many studies 25, 26, 27. Both factors are integral components of the TNM classification 28. They are visible signs of advanced local extension and connection to the vascular system as a requirement for distant spread. The additional factors invasion type and tumor budding are well known, but did not achieve recommendation in national guidelines 2, 29. The infiltrative invasion type is associated with aggressive behavior 30. However, the interobserver agreement is less than optimal 13 and it seems that difficulties in interpreting the histological features from Jass’ method led to an overdiagnosis of this feature with a loss of prognostic strength. In our opinion, this invasion type occurs in no more than 10% to 15% of cases (Fig. 1B). Tumor budding has been investigated in many studies as a prognostic factor for tumors of the gastrointestinal tract 31. Unfortunately, this feature is hampered by several different competing definitions, cutoffs, and methods of evaluation. A recently performed study revealed only a fair interobserver agreement among 10 investigators 32. The interobserver agreement in this study was substantial with a *κ*‐value of 0.61. Nevertheless, regardless of the way tumor budding is judged, the vast majority of studies confirmed an association between tumor budding and other worse factors such as lymph node metastases and aggressive clinical course 31. Tumor budding is believed to reflect epithelial mesenchymal transition which promotes tumor cell migration and tumor progression 33. The WNT/Wingless signaling pathway is involved in this process of epithelial mesenchymal transition by influencing *β*‐catenin and E‐cadherin 34, 35. Therefore, it seems to be a relevant morphologic adjunctive factor that reflects a certain molecular feature that promotes tumor progression. Lymph node size is the most recently introduced prognostic factor and is associated with lymph node yield and outcome 11, 36. Our group demonstrated that the paucity of lymph nodes with diameters >5 mm, termed LN5vl, is an independent prognostic factor in conventional lymph node dissected colon cancer 10 and after employment of advanced dissection techniques. We believe that this factor can serve as a surrogate marker for an impaired immune response 37. Along with the fact that LN5vl is extremely easy to determine, this makes it an ideal complement to previously established factors. A problem of NCCN risk factor stratification could be that a high proportion of tumors show at least one worse feature. In our analysis, 81 of 165 stage II cancers fell into this category. Compared to a rate of 10% to 20% of cases that show an aggressive course, this proportion seems to be too high. The MBR high‐risk group comprises only 10% of stage I/II cases and 17% of stage II cancers which seem more appropriate. In comparison to the NCCN risk stratification, its prognostic strength is higher (Fig. 3C and 3E).

Without a doubt, morphological stratification is hampered by the subjectivity of the investigator. On the other hand, it is unbeatable regarding costs, efforts, and availability. It is an interesting question whether the future of cancer stratification will be molecular or morphologic. It could be a smart approach to combine the best of two worlds. Such an attempt has already been made by Srivastava et al. in hepatocellular carcinoma 38.

In conclusion, the MBR score includes morphological factors that reflect the extension of local spread, angioinvasion, epithelial mesenchymal transition, aggressive phenotype, and immune response. In this retrospective analysis, the MBR score was the only independent prognostic factor regarding overall survival in stage I/II colon cancers. Future clinical consequences that could be drawn are that an intensified surveillance could be recommended for the patients of the intermediate‐risk group. However, patients of the high‐risk group could benefit from adjuvant chemotherapy. We are currently planning to perform a multicenter study restricted to stage II colon cancer to verify the findings of this study in a prospective design.

To answer the question of whether these high‐risk patients could benefit from adjuvant chemotherapy, a considerably larger study with a prospective design is necessary.

## Conflict of Interest

All authors declare that no conflict of interests exists.

## References

[1] American Cancer Society . 2014 Colorectal Cancer Facts & Figures 2014‐2016. American Cancer Society, Atlanta.

[2] NCCN Clinical Practice Guidelines in Oncology (NCCN Guidelines) . 2015 in Colon Cancer (Version 2.2015). Available at: http://www.nccn.org/professionals/physician_gls/f_guidelines.asp

[3] Benson, A. B. III , D. Schrag , M. R. Somerfield , A. M. Cohen , A. T. Figueredo , P. J. Flynn , et al. 2004 American Society of Clinical Oncology recommendations on adjuvant chemotherapy for stage II colon cancer. J. Clin. Oncol. 22:3408–3419. 15199089 10.1200/JCO.2004.05.063

[4] Goldstein, N. S. 2002 Lymph node recoveries from 2427 pT3 colorectal resection specimens spanning 45 years: recommendations for a minimum number of recovered lymph nodes based on predictive probabilities. Am. J. Surg. Pathol. 26:179–189. 11812939 10.1097/00000478-200202000-00004

[5] Swanson, R. S. , C. C. Compton , A. K. Stewart , and K. I. Bland . 2003 The prognosis of T3N0 colon cancer is dependent on the number of lymph nodes examined. Ann. Surg. Oncol. 10:65–71. 12513963 10.1245/aso.2003.03.058

[6] Gray, R. G. , P. Quirke , K. Handley , M. Lopatin , L. Magill , F. L. Baehner , et al. 2011 Validation study of a quantitative multigene reverse transcriptase‐polymerase chain reaction assay for assessment of recurrence risk in patients with stage II colon cancer. J. Clin. Oncol. 29:4611–4619. 22067390 10.1200/JCO.2010.32.8732

[7] Maak, M. , I. Simon , U. Nitsche , P. Roepman , M. Snel , A. M. Glas , et al. 2013 Independent validation of a prognostic genomic signature (ColoPrint) for patients with stage II colon cancer. Ann. Surg. 257:1053–1058. 23295318 10.1097/SLA.0b013e31827c1180

[8] Pages, F. , J. Galon , M. C. Dieu‐Nosjean , E. Tartour , C. Sautes‐Fridman , and W. H. Fridman . 2010 Immune infiltration in human tumors: a prognostic factor that should not be ignored. Oncogene 29:1093–1102. 19946335 10.1038/onc.2009.416

[9] Galon, J. , B. Mlecnik , G. Bindea , H. K. Angell , A. Berger , C. Lagorce , et al. 2014 Towards the introduction of the ‘Immunoscore’ in the classification of malignant tumours. J. Pathol. 232:199–209. 24122236 10.1002/path.4287 PMC4255306

[10] Märkl, B. , T. Schaller , Y. Kokot , K. Endhardt , H. Kretsinger , K. Hirschbühl , et al. 2015 Lymph node size as a simple prognostic factor in node negative colon cancer and an alternative thesis to stage migration. Am. J. Surg. doi:10.1016/j.amjsurg.2015.05.026 [Epub ahead of print]. 10.1016/j.amjsurg.2015.05.026 26307422

[11] Märkl, B. , J. Rössle , H. M. Arnholdt , T. Schaller , I. Krammer , C. Cacchi , et al. 2012 The clinical significance of lymph node size in colon cancer. Mod. Pathol. 25:1413–1422. 22684222 10.1038/modpathol.2012.92

[12] Märkl, B. , I. Renk , D. V. Oruzio , H. Jähnig , G. Schenkirsch , C. Scholer , et al. 2010 Tumour budding, uPA and PAI‐1 are associated with aggressive behaviour in colon cancer. J. Surg. Oncol. 102:235–241. 20740581 10.1002/jso.21611

[13] Jass, J. R. , Y. Ajioka , J. P. Allen , Y. F. Chan , R. J. Cohen , J. M. Nixon , et al. 1996 Assessment of invasive growth pattern and lymphocytic infiltration in colorectal cancer. Histopathology 28:543–548. 8803598 10.1046/j.1365-2559.1996.d01-467.x

[14] Schemper, M. , and T. L. Smith . 1996 A note on quantifying follow‐up in studies of failure time. Control. Clin. Trials 17:343–346. 8889347 10.1016/0197-2456(96)00075-x

[15] Dekervel, J. , D. Hompes , H. van Malenstein , D. Popovic , X. Sagaert , B. De Moor , et al. 2014 Hypoxia‐driven gene expression is an independent prognostic factor in stage II and III colon cancer patients. Clin. Cancer Res. 20:2159–2168. 24486594 10.1158/1078-0432.CCR-13-2958

[16] Jass, J. R. 2007 Classification of colorectal cancer based on correlation of clinical, morphological and molecular features. Histopathology 50:113–130. 17204026 10.1111/j.1365-2559.2006.02549.x

[17] Guinney, J. , R. Dienstmann , X. Wang , A. de Reynies , A. Schlicker , C. Soneson , et al. 2015 The consensus molecular subtypes of colorectal cancer. Nat. Med. 21:1350–1356. 26457759 10.1038/nm.3967 PMC4636487

[18] Pages, F. , A. Kirilovsky , B. Mlecnik , M. Asslaber , M. Tosolini , G. Bindea , et al. 2009 In situ cytotoxic and memory T cells predict outcome in patients with early‐stage colorectal cancer. J. Clin. Oncol. 27:5944–5951. 19858404 10.1200/JCO.2008.19.6147

[19] Figueredo, A. , M. L. Charette , J. Maroun , M. C. Brouwers , and L. Zuraw . 2004 Adjuvant therapy for stage II colon cancer: a systematic review from the Cancer Care Ontario Program in evidence‐based care's gastrointestinal cancer disease site group. J. Clin. Oncol. 22:3395–3407. 15199087 10.1200/JCO.2004.03.087

[20] Gill, S. , C. L. Loprinzi , D. J. Sargent , S. D. Thome , S. R. Alberts , D. G. Haller , et al. 2004 Pooled analysis of fluorouracil‐based adjuvant therapy for stage II and III colon cancer: who benefits and by how much? J. Clin. Oncol. 22:1797–1806. 15067028 10.1200/JCO.2004.09.059

[21] Quasar Collaborative, G. , R. Gray , J. Barnwell , C. McConkey , R. K. Hills , N. S. Williams , et al. 2007 Adjuvant chemotherapy versus observation in patients with colorectal cancer: a randomised study. Lancet 370:2020–2029. 10.1016/S0140-6736(07)61866-2 18083404

[22] O'Connor, E. S. , D. Y. Greenblatt , N. K. LoConte , R. E. Gangnon , J. I. Liou , C. P. Heise , et al. 2011 Adjuvant chemotherapy for stage II colon cancer with poor prognostic features. J. Clin. Oncol. 29:3381–3388. 21788561 10.1200/JCO.2010.34.3426 PMC3164243

[23] Figueredo, A. , M. E. Coombes , and S. Mukherjee . 2008 Adjuvant therapy for completely resected stage II colon cancer. Cochrane Database Syst. Rev. doi:10.1002/14651858.CD005390.pub2 10.1002/14651858.CD005390.pub2 PMC8885310 18646127

[24] Benson, A. B. III . 2006 New approaches to the adjuvant therapy of colon cancer. Oncologist 11:973–980. 17030637 10.1634/theoncologist.11-9-973

[25] O'Connell, J. B. , M. A. Maggard , and C. Y. Ko . 2004 Colon cancer survival rates with the new American Joint Committee on Cancer sixth edition staging. J. Natl Cancer Inst. 96:1420–1425. 15467030 10.1093/jnci/djh275

[26] Gibson, K. M. , C. Chan , P. H. Chapuis , O. F. Dent , and L. Bokey . 2014 Mural and extramural venous invasion and prognosis in colorectal cancer. Dis. Colon Rectum 57:916–926. 25003286 10.1097/DCR.0000000000000162

[27] Huh, J. W. , J. H. Lee , H. R. Kim , and Y. J. Kim . 2013 Prognostic significance of lymphovascular or perineural invasion in patients with locally advanced colorectal cancer. Am. J. Surg. 206:758–763. 23835209 10.1016/j.amjsurg.2013.02.010

[28] Sobin L. H., Gospodarowicz M. K., and Wittekind C., eds. 2010 TNM classification of malignant tumours. John Wiley & Sons Ltd, West Sussex.

[29] Pox, C. , S. Aretz , S. C. Bischoff , U. Graeven , M. Hass , P. Heussner , et al. 2013 [S3‐guideline colorectal cancer version 1.0]. Z. Gastroenterol. 51:753–854. 23955142 10.1055/s-0033-1350264

[30] Jass, J. R. , S. B. Love , and J. M. Northover . 1987 A new prognostic classification of rectal cancer. Lancet 1:1303–1306. 2884421 10.1016/s0140-6736(87)90552-6

[31] Märkl, B. , and H. M. Arnholdt . 2011 Prognostic significance of tumor budding in gastrointestinal tumors. Expert Rev. Anticancer Ther. 11:1521–1533. 21999126 10.1586/era.11.156

[32] Puppa, G. , C. Senore , K. Sheahan , M. Vieth , A. Lugli , I. Zlobec , et al. 2012 Diagnostic reproducibility of tumour budding in colorectal cancer: a multicentre, multinational study using virtual microscopy. Histopathology 61:562–575. 22765314 10.1111/j.1365-2559.2012.04270.x

[33] Zlobec, I. , and A. Lugli . 2010 Epithelial mesenchymal transition and tumor budding in aggressive colorectal cancer: tttumor budding as oncotarget. Oncotarget 1:651–661. 21317460 10.18632/oncotarget.199 PMC3248128

[34] Brabletz, T. , A. Jung , S. Reu , M. Porzner , F. Hlubek , L. A. Kunz‐Schughart , et al. 2001 Variable beta‐catenin expression in colorectal cancers indicates tumor progression driven by the tumor environment. Proc. Natl. Acad. Sci. USA. 98:10356–10361. 11526241 10.1073/pnas.171610498 PMC56965

[35] Brabletz, T. , F. Hlubek , S. Spaderna , O. Schmalhofer , E. Hiendlmeyer , A. Jung , et al. 2005 Invasion and Metastasis in Colorectal Cancer: Epithelial‐Mesenchymal Transition, Mesenchymal‐Epithelial Transition, Stem Cells and &beta;‐Catenin. Cells Tissues Organs 179:56–65. 15942193 10.1159/000084509

[36] Okada K, Sadahiro S , Suzuki T , A. Tanaka , G. Saito , S. Masuda , et al. 2015 The size of retrieved lymph nodes correlates with the number of retrieved lymph nodes and is an independent prognostic factor in patients with stage II colon cancer. Int. J. Colorectal Dis. 12:1685–1693. 26260481 10.1007/s00384-015-2357-9 PMC4675793

[37] Märkl, B. 2015 Stage migration versus immunology – the lymph node count story in colon cancer. World J. Gastroenterol. 21:12218–12233. 26604632 10.3748/wjg.v21.i43.12218 PMC4649108

[38] Srivastava, S. , K. F. Wong , C. W. Ong , C. Y. Huak , K. G. Yeoh , M. Teh , et al. 2012 A morpho‐molecular prognostic model for hepatocellular carcinoma. Br. J. Cancer 107:334–339. 22713659 10.1038/bjc.2012.230 PMC3394972

